# Supplementary material for: Development of the core outcome set for herbal medicine treatment of lumbar disc herniation (COS-HM-LDH): A study protocol for a systematic review and a delphi survey
Source: PLoS One. 2025 Nov 7;20(11):e0335712. doi: 10.1371/journal.pone.0335712 (PMC12594413; doi:10.1371/journal.pone.0335712)
Supplement: S1 Table — (DOC) [file pone.0335712.s001.doc]

**S1 Table.** Search strategy for English databases

| **Search strategy** |
| --- |
| **Database PubMed** |
| #1 "lumbarised"[All Fields] OR "lumbarization"[All Fields] OR "lumbarized"[All Fields] OR "lumbars"[All Fields] OR "lumbosacral region"[MeSH Terms] OR ("lumbosacral"[All Fields] AND "region"[All Fields]) OR "lumbosacral region"[All Fields] OR "lumbar"[All Fields]) AND ("intervertebral disc displacement"[MeSH Terms] OR ("intervertebral"[All Fields] AND "disc"[All Fields] AND "displacement"[All Fields]) OR "intervertebral disc displacement"[All Fields] OR ("disc"[All Fields] AND "herniation"[All Fields]) OR "disc herniation"[All Fields] |
| #2 "plants, medicinal"[MeSH Terms] OR ("plants"[All Fields] AND "medicinal"[All Fields]) OR "medicinal plants"[All Fields] OR ("herbal"[All Fields] AND "medicines"[All Fields]) OR "herbal medicines"[All Fields] OR "plant extracts"[MeSH Terms] OR ("plant"[All Fields] AND "extracts"[All Fields]) OR "plant extracts"[All Fields] OR "herb*"[All Fields] OR ("natural"[All Fields] AND "products"[All Fields]) OR "natural products"[All Fields] |
| #3 (#1 AND #2) |
| #4 Filters: Humans |
| **Database Cochrane Library** |
| #1 MeSH descriptor: [Intervertebral Disc Displacement] explode all trees |
| #2 MeSH descriptor: [Herbal Medicine] explode all trees |
| #3 MeSH descriptor: [Herb-Drug Interactions] explode all trees |
| #4 MeSH descriptor: [Phytotherapy] explode all trees |
| #5 MeSH descriptor: [Biological Products] explode all trees |
| #6 MeSH descriptor: [Plant Extracts] explode all trees |
| #7 (#2 or #3 or #4 or #5 or #6) |
| #8 lumbar disc herniation or herniated disc or intervertebral disc displacement |
| #9 (#1 or #8) |
| #10 herbal or natural products or plant extracts |
| #11 (#7 or #10) |
| #12 (#9 and #11) |
