# Supplementary material for: Development of the core outcome set for herbal medicine treatment of lumbar disc herniation (COS-HM-LDH): A study protocol for a systematic review and a delphi survey
Source: PLoS One. 2025 Nov 7;20(11):e0335712. doi: 10.1371/journal.pone.0335712 (PMC12594413; doi:10.1371/journal.pone.0335712)
Supplement: Attachment — Submitted filename: response to reviewers.docx [file pone.0335712.s003.docx]

**Editor’s Comments**

- In this study, the authors intend to develop a Core Outcome Set for Herbal Medicine Treatment of Lumbar Disc Herniation (COS-HM-LDH). However, the methodology section is entirely in the future tense, indicating that the proposed steps have yet to be established and standardized.
- This raises questions about the study's foundation; it seems to focus on potential future actions rather than presenting a concrete protocol.
- To enhance clarity, the authors should address this concern directly, providing a rationale for their approach and the relevance of discussing future methodologies.
- An expanded discussion on the purpose of the study and its significance in the context of existing literature would also strengthen their argument.
- This would help clarify the study's objectives and the importance of developing standardized outcomes for herbal treatments in this area.

Response:

- Thank you for your thoughtful comments. In response, we have revised the methodology section by changing the tense from future to present to clearly reflect the protocol nature of the study and to distinguish between ongoing and completed components. Additionally, we have expanded the discussion section to better articulate the significance of this study in the context of existing literature, emphasizing the current lack of standardized outcome measures for herbal medicine treatment of LDH and how this study addresses that gap. Revised parts have been marked in red in the manuscript.

**Reviewer 1 - Accept**

Well constructed manuscript. Looking forward for your valuable contribution to the scientific community.

Response:

- We sincerely thank the reviewer for their positive and encouraging feedback.

**Reviewer 2 - Accept**

Well-articulated, and a good initiative to establish standard guidelines. This study can serve as a guiding document for any further works based on complementary and alternative medicine, globally.

Response:

- We are grateful for the reviewer’s kind words and appreciation of our efforts. We hope this study will contribute meaningfully to future standardization in herbal medicine research.

**Reviewer 3 – Minor Revision**

Table 1 Patients or Their Representatives – why this data is originally not collected?, Graphical abstract is required.

Response:

- We appreciate the reviewer’s valuable suggestions. In this study, we aimed to reflect patient perspectives by incorporating data from a previously published nationwide survey that investigated the real-world symptoms and needs of patients with LDH (Kim et al., 2023), rather than recruiting patients or their representatives directly. This approach enabled us to ensure the inclusion of patient-centered outcomes while avoiding duplication of efforts and addressing feasibility constraints within the current research scope. To further enhance the clarity and accessibility of the study, we have also created and included a graphical abstract that visually summarizes the background, aim, methodological phases, and expected impact of the COS-HM-LDH development.
