# Supplementary material for: Development of the core outcome set for herbal medicine treatment of lumbar disc herniation (COS-HM-LDH): A study protocol for a systematic review and a delphi survey
Source: PLoS One. 2025 Nov 7;20(11):e0335712. doi: 10.1371/journal.pone.0335712 (PMC12594413; doi:10.1371/journal.pone.0335712)
Supplement: Attachment — Submitted filename: Response to reviewers 3.docx [file pone.0335712.s005.docx]

We appreciate the thoughtful review and helpful comments. We have addressed each of the comments here and in the manuscript.

**Journal Requirements**

Comment: Your ethics statement should only appear in the Methods section of your manuscript. If your ethics statement is written in any section besides the Methods, please delete it from any other section.

Response: Thank you for this important reminder. We have removed the “Ethics declarations” section from the end of the manuscript and relocated the corresponding content to the Methods section under a new subsection titled “Ethical approval.” This revision ensures that the ethics statement appears only in the Methods section, in line with the journal’s requirements.

**Revised Sentence (Page 13):**
*“Ethical approval to conduct this study has been granted by The Institutional Review Board of the Korea Institute of Oriental Medicine (IRB approval no. I-2312/012-003-04). All individuals participating in the Delphi rounds will provide informed consent prior to their involvement in the study.”*

**Reviewer #4**

**Comment 1:** Background: offers clear rationale to the study, highlighting the need for establishing a COS to evaluate the effectiveness of herbal medicine in lumbar disc herniation management.

**Response:** We thank the reviewer for this positive feedback.

**Comment 2:** Methodology – The four-phase development process (systematic review, Delphi surveys with experts and clinicians, and a consensus meeting) is well organized and follows established COS development frameworks. The consensus method is described here a priori. The figure offers visual summary of the proposed protocol.
**Response:** We thank the reviewer for this positive and encouraging feedback on our methodological framework.

**Comment 3:** Development of the Initial COS List: this step is not very clear. What is the criteria that will be used to synthesis the initial set of COS? Will the PMG employ theme saturation or another qualitative research method? The patients’ perspective should be considered in this step, as per standard 8 of COS-STAD.

**Response:** Thank you for this important comment. We have clarified that the PMG will include all outcomes from the systematic review, merge similar items, and report their frequency across studies to form the initial COS list. To meet COS-STAD standard 8, we also incorporated findings from a nationwide patient survey, ensuring that patient-prioritized symptoms and treatment needs are reflected in the initial questionnaire for the Delphi process.

**Revised Sentence (Page 7-8):**
*“After reviewing the collected outcomes and EMs, the PMG refines and finalizes a list consistent with the objectives of this COS. All outcomes extracted from the systematic review are included in the initial list to ensure comprehensiveness. For each outcome, the number of studies in which it was reported will also be presented, thereby providing information on the frequency and strength of use in previous research. In addition, to reflect patients’ perspectives, findings from the previously conducted nationwide survey of LDH patients are incorporated, highlighting patient-prioritized symptoms and treatment needs [25]. These combined sources form the basis of the initial COS questionnaire, which will be used for the first Delphi process.”*

**Comment 4:** The authors note that patient participation has been replaced by reference to a previously published patient survey on the same topic. Including a brief summary of this survey in the introduction would enhance the manuscript’s value.
**Response:** As suggested, we have added a concise summary of the nationwide patient survey in the Background section, highlighting priority symptoms (e.g., back pain, leg pain, functional disability) and treatment preferences (e.g., safety and stable effect).

**Revised Sentence (Page 4-5):**
*“To address these concerns, a recent nationwide web-based survey of 500 LDH patients in South Korea investigated their real-world symptoms and priorities [25]. The most common symptoms reported were leg numbness (87.0%) and back pain (81.2%), while the most common disabilities were discomfort in sitting (64.6%) and lifting (63.6%). Back pain (48.4%) was identified as the highest priority symptom for improvement. Importantly, a majority of patients expressed preference for improvement in disability over pain (55.8%), a stable effect over a rapid effect (78.2%), and safety over treatment efficacy (56.4%). Safety (25.8%) and cost (22.2%) were also recognized as important treatment factors. Based on these findings, we aim to identify the essential outcome measures that should be prioritized in LDH management.”*

**Comment 5**: Furthermore, can the survey findings be objectively integrated into the Delphi process outcomes? If not, the inclusion of patient representatives in the Delphi rounds perhaps should be considered?

**Response:** Thank you for this helpful comment. We revised the Methods section to state that the findings from the nationwide patient survey have been incorporated into the development of the initial COS list. These patient-prioritized outcomes form part of the first Delphi questionnaire, ensuring that patient perspectives are objectively reflected in the Delphi process.

**Revised Sentence (Page 7-8):**
*“…In addition, to reflect patients’ perspectives, findings from the previously conducted nationwide survey of LDH patients are incorporated, highlighting patient-prioritized symptoms and treatment needs [25]. These combined sources form the basis of the initial COS questionnaire, which will be used for the first Delphi process.”*

**Comment 6:** I commend the authors for their thorough and well-structured approach and hope to see the results of their effort published out soon.
**Response:** We sincerely thank the reviewer for the encouraging comments.

We are encouraged by this feedback and will strive to ensure that the study produces meaningful results.
